# A Case of Strangulation of the Posterior Lip of the Os Tincœ, within the Superior Aperture of the Pessary

**Published:** 1855-06

**Authors:** C. Goodbrake

**Affiliations:** Clinton, Ill.


ARTICLE II.—*A Case of Strangulation of the posterior lip of the Os Tincæ, within the superior aperture of the Pessary.* By C. Goodbrake, M. D., Clinton, Ill.

Editors of the Journal:—The use of pessaries of all sorts of material, and of almost every imaginable form and size, has become so fashionable, that a description of the following case may not prove entirely uninteresting to the readers of the Journal:

About two years since I was called to see a Mrs. B., in consultation with my friend, Dr. Warner, and upon arriving at the bedside, we received from the lady the following history of her case:

She informed us that she had suffered from ill health for some time, and that about five months previous to our visit, she had consulted a doctor, who informed her that she had prolapsus uteri, and that he could cure her without any difficulty by introducing an instrument into the vagina. The woman anxious to get well of course submitted to the introduction, and nothing more was done for the space of *five* months. At the end of this time she requested the doctor to remove it, as she did not receive any benefit, but on the contrary, suffered a great deal of inconvenience from its use. The doctor then endeavored to remove it, but found to his utter astonishment that he was unable (as the woman said) to budge it. The doctor made several efforts from day to day, to remove it, but without success. He then informed the woman that she would have to make herself content, until such time as he could procure a speculum, for the purpose of *seeing into* the nature of the difficulty.

But in the mean time, the lady (as well as her husband) becoming very anxious to have the artificial obstruction removed if possible, Dr. Warner and myself were called in.

Upon examination we found the instrument in question to be a hollow glass pessary, in the form of a flattened globe, with an aperture through its shorter axis. Also that the posterior lip of the os tincæ, had from some cause or other descended into the superior opening of the pessary, and had become strangulated. The pessary could very easily be made to rotate upon the neck of the strangulated portion, but all our efforts to remove it proved unuccessful.

At length, after trying every other method we could think of, without success, I hit upon the following plan: I introduced two fingers over the pessary, one finger on either side of the strangulated portion, and pressed very gently but steadily downwards, and in the course of a few minutes, I was enabled, without much pain to the patient, to bring the pessary without the os externum. (I may as well state here that the strangulated portion inside the pessary, had enlarged to the size of a small hickory nut, and that the balance of the pessary was filled up with a thick, dark and very offensive fluid.)

I next procured a piece of soft buckskin, and cut a longitudinal slit in it,—similar to a retractor,—I placed this between the vulva of the patient and the pessary, so as completely to protect the external organs of generation, and also to prevent any of the glass from being drawn into the vagina. I then held a piece of metal beneath the pessary, and struck it a blow with a hammer, which broke it to pieces and allowed the womb to return to its proper position; and the patient was relieved from her very disagreeable condition. By remaining in the recumbent position, and using proper applications,—by means of the female syringe—for about a week, the lady got along without any farther trouble.

One object in writing this article, was to caution the profession against the use of the peculiar kind of pessary, a description of which has already been given; as the case proves that disagreeable consequences may follow. And I would here say, that in my opinion the indiscriminate use of the pessary, is a very injurious and highly reprehensible practice; and that leaving them in *situ* for months ought not to be thought of. Indeed I am free to say, that so far as my experience and observation extend, cases are very rare where the use of the pessary is followed by any permanent benefit to the patient; but on the contrary,—taking the anatomy of the parts, as well as the causes and nature of prolapsus uteri into consideration, the introduction of a foreign body into the vagina, for the the purpose of keeping the uterus in its proper position, is, in nine cases out of ten, followed by more disappointments than good results.

